# The application of the cytokinesis-block micronucleus assay on peripheral blood lymphocytes for the assessment of genome damage in long-term residents of areas with high radon concentration

**DOI:** 10.1093/jrr/rrt091

**Published:** 2013-08-01

**Authors:** Maxim Yu. Sinitsky, Vladimir G. Druzhinin

**Affiliations:** 1 Department of Genetics, Kemerovo State University, Krasnaya Str 6, Kemerovo, 650043, Russian Federation; 2 Institute of Human Ecology of SB RAS, Leningradsky Ave 10, Kemerovo, 650065, Russian Federation

**Keywords:** micronucleus assay, micronuclei, genotoxicity, cytochalasin B, ionising radiation, radon, children

## Abstract

Estimating the effects of small doses of ionising radiation on DNA is one of the most important problems in modern biology. Different cytogenetic methods exist to analyse DNA damage; the cytokinesis-block micronucleus assay (CBMN) for human peripheral blood lymphocytes is a simple, cheap and informative cytogenetic method that can be used to detect genotoxic-related markers. With respect to previous studies on radiation-induced genotoxicity, children are a poorly studied group, as evidenced by the few publications in this area. In this study, we assessed radon genotoxic effects by counting micronuclei (MN), nucleoplasmic bridges (NPBs) and nuclear buds (NBUDs) in the lymphocytes of children who are long-term residents from areas with high radon concentrations. In the exposed group, radon was found to cause significant cytogenetic alterations. We propose that this method can be employed for biomonitoring to screen for a variety of measures.

## INTRODUCTION

Estimating the biomedical long-term effect of small doses of ionising radiation is a complicated issue that affects not only radiobiology but also social and economic spheres. It has been reported that > 60% of the ionising radiation a person receives every year can be caused by natural sources of radiation, and more than 50% of this radiation can be due to radon and the products of its disintegration. Therefore, maintaining radon safety is one of the most critical challenges in ecology, and this has been actively discussed throughout the last few decades [1]. A correlation between radon and the frequency of lung cancer has been confirmed in recent years by extensive epidemiological research. However, it is important to note that current models estimating the risk of radiation-related hazards are based upon analysis of data collected from irradiated miners. Currently, it is not clear how transferrable this risk model is to studies involving inhabitants of areas with high-radiation conditions [8, 12]. Therefore, assessing the effects of radon in areas that have a developed mining industry is of particular interest. The mining region of Kemerovo is located in the Russian Federation [2].

In addition to using methods of dosimetry to evaluate the risk of harmful radiation effects, the severity of such a risk should be evaluated by a biological marker. Biological test systems can evaluate the risks of radiation and provide a thorough assessment of the quality of the environment and its suitability for humans [2]. In 1983, Hsu hypothesised that environmental exposure can induce chromosome damage, varying along a continuum in the general population; higher levels would be observed in individuals with an inherent susceptibility to DNA damage. Hsu *et al*. (1989) developed a mutagen sensitivity assay to detect potential variations in susceptibility to the effects of mutagenic agents [10, 11]. Each genetic alteration or mutation, whether an initiating or a progression-associated event, can cause gross chromosomal changes and can therefore be detected cytogenetically [3, 14]. In the classical cytogenetic techniques, chromosomes are studied directly by observation and by counting the aberrations in metaphase spreads. This approach provides the most detailed analysis, but the complexity and laboriousness of enumerating the aberrations in metaphase spreads, confounded by an artefactual loss of chromosomes from metaphase preparations has prompted the development of a simpler system for measuring chromosome damage [15].

A method of mapping cytogenetic effects in a population within a large industrial region has been developed and used by the staff of the Department of Genetics, Kemerovo State University. This method has yielded results from a 15-year study involving the Kemerovo region, which is characterised by unfavourable ecological and genetic circumstances. In particular, an unusually high frequency of aberrant metaphase spreads (5.78 ± 0.63%) was found in 1992 among a sample of children and adolescents living in the southern part of the Kemerovo region, namely Gornaya Shoria (the Tashtagolsky district). The absence of significant chemical pollutants in this region, along with a prevalence of dicentric and ring chromosomes (0.32 ± 0.05%) suggests that these clastogenic effects stem from radiation. The Kemerovo study prompted further cytogenetic analysis of the population residing in this region [2].

Schmid [15] and Heddle [9] independently proposed an alternative and simpler approach for assessing chromosome damage *in vivo* and that was measuring micronuclei (MN), also known as Howell–Jolly bodies. The cytokinesis-block micronucleus assay (CBMN) in bone marrow and peripheral blood lymphocytes is now one of the most established *in vivo* cytogenetic assays in the field of genetic toxicology. However, this technique is not applicable to other cell populations *in vivo* or *in vitro*, and other methods have since been developed to measure MN in a variety of nucleated cell types *in vitro* [13]. MN are found in dividing cells that either contain chromosome breaks lacking centromeres (acentric fragments) and/or whole chromosomes that are unable to travel to the spindle poles during mitosis. By telophase, a nuclear envelope is formed around the lagging chromosomes and fragments, which then uncoil and gradually assume the morphology of an interphase nucleus with the exception that they are smaller than the main nucleus in the cell; hence, they are called “micronuclei.” MN, therefore, provide a convenient and reliable index of both chromosome breakage and chromosome loss. Because MN are expressed in cells that have completed nuclear division, they can be ideally scored during the binucleated stage of the cell cycle [7]. Occasionally, nucleoplasmic bridges (NPBs) are observed between the nuclei in a binucleated cell. These are most likely dicentric chromosomes in which the two centromeres have been pulled to opposite poles of the cell, and the DNA in the resulting bridge has been covered by nuclear membrane. Thus, NPBs in binucleated cells provide an additional and complementary measure of chromosome rearrangement, which can be scored together with the micronuclei count [4]. In addition to MN and NPBs, the CBMN assay detects nuclear buds (NBUDs) or protrusions, which represent a mechanism by which cells remove amplified DNA; they are therefore considered markers of possible gene amplification [5].

The CBMN test is slowly replacing the analysis of chromosome aberrations in lymphocytes because the damaged cytogenetic markers are easy to recognise and score and the results can be obtained in a shorter time. In addition, information regarding other cellular events such as mitotic rate and cell death by apoptosis and necrosis can be simultaneously obtained from the same slides [6].

The purpose of this research is to use a biological test system based on the CBMN on human peripheral blood lymphocytes to measure genome damage in people residing in conditions of high radon concentrations.

## MATERIALS AND METHODS

Blood samples were obtained from 60 children and teenagers, 8–17 years old, who had been residing long-term (3.01 ± 0.05 years) at a boarding school located in the Tashtagolsky district of the Kemerovo region. This area has low levels of air pollution from chemical compounds that can induce genetic damage. Dietary studies of these children did not reveal any factors with known mutagenic capacity. However, radon levels measured in the educational and living quarters of the boarding school were high (626.0 Bq/m^3^) (Table 1).

**Table 1.** Radon concentration in the rooms of the boarding school (Tashtagol city, exposed group) and of the control settlement (Zarubino village)

Settlement	Date of measurement	Number of measuring points	Average unit volume activity of radon, Bq/m^3^, M ± m	Limit variation, Bq/m^3^–Bq/m^3^,
Tashtagol	11 Feb 2011	10	905 ± 134	650–1143
Tashtagol	2 Mar 2011	18	347 ± 49	74–749
Zarubino	25 Jan 2011	10	64 ± 13	39–203
Zarubino	6 Apr 2011	17	119 ± 27	53–172

Adolescents are an optimal study group for this research because influences from unhealthy habits, diseases and occupational exposures are significantly minimal at this age. In addition to living in the same area, all members in this study had similar diets and accommodation. For each group, a protocol was designed and an informed consent form was signed by the parents or guardians. Detailed descriptions of these are presented in Table 2.

**Table 2.** Gender and age of children/teenagers included in the exposed group

Person	Number	Age (M ± m)	Limit variation
Total	60	12.1 ± 0.31	8–17
Male	34	12.1 ± 0.45	8–16
Female	26	12.0 ± 0.48	8–17

Children living in the Zarubino village (Kemerovo region) constituted a control group. This area lacks chemical contaminants and has low radon levels (91.5 Bq/m^3^) (Table 1). Characteristics of this group are presented in Table 3.

**Table 3.** Gender and age of children/teenagers included in the control group

Person	Number	Age (M ± m)	Limit variation
Total	60	14.9 ± 0.32	8–18
Male	27	14.8 ± 0.42	9–17
Female	33	15.0 ± 0.47	8–18

Blood from the adolescents and children was sampled using vacutainers with heparin before culturing and was stored at 4°C for 24 h. The blood (200 µl) was then transferred to flasks containing 3.8 ml of culture medium (RPMI-1640 + 20% inactivated bovine serum + 100 U/ml ampicillin). Based on an assessment of an adaptive response, three cultures were created from each sample. Phytohaemagglutinin (PHA) (30 mg/flask) was added to each culture and the flasks were incubated for 44 h at 37°C. After a 44-h incubation period, 6 µg/ml of cytochalasin B was poured into each culture and allowed to incubate another 24 h at 37°C. The cells were then resuspended in the flasks, poured into centrifuge tubes and centrifuged for 10 min at 1000 rev/min. The supernatant was removed, the pellet was broken and 1 ml of cold, freshly prepared 0.125 M KCl was poured onto the wall of the tube. The pellet was gently resuspended in the KCl solution and another 4 ml was added. The tube was then closed and inverted several times (for ∼ 30 s). After the pellet was resuspended, 1 ml of cold freshly prepared Carnoy's fixer (a compound of methanol and glacial acetic acid in a ratio of 3:1) was poured onto the wall of the tube and stirred. The samples were stored at − 20°C until the next centrifugation step. The suspension was centrifuged for 10 min at 1000 rev/min. The supernatant was removed, the pellet was broken. Without inverting, another 5 ml of cold fixer (a compound of methanol and glacial acetic acid in the ratio of 3:1) was poured onto the pellet. This procedure was repeated several times until the pellet appeared clean and the cell suspension was clear.

After the last centrifugation step, the majority of the supernatant was removed, leaving a volume not exceeding 200 µl.

Next, the suspension was gently transferred to a dry, cold glass slide using a pipette. Azure-eosin staining in a phosphate buffer was carried out for 15 min. The slides were analysed using a Nikon Eclipse 80i microscope with transmitted light and a full filter at × 800–1000 magnification (oil immersion). We used the following criteria to identify the micronuclei:

- the diameter of a MN in human lymphocytes usually ranges from 1/16th to 1/3rd of the mean diameter of the main nuclei which corresponds to 1/256th and 1/9th of the area of one of the main nuclei in a BN cell, respectively;
- MN are non-refractile and they can therefore be distinguished from artefacts such as staining particles;
- MN are not linked or connected to the main nuclei;
- MN may touch but not overlap the main nuclei, and the micronuclear boundary should be distinguishable from the nuclear boundary;
- MN usually have the same staining intensity as the main nuclei, but occasionally staining may be more intense [4].

Statistical analysis of the study was performed using the program BioStat 2009, Professional 5.8.4 with a block of nonparametric statistics.

## RESULTS AND DISCUSSION

For each sample, 500 cells were counted to determine the spectrum of phenotypes. Cells were observed in the preparations to have varying numbers of nuclei. The presence of a mononuclear cell suggested that the cell had not responded to a mitotic signal and was not undergoing mitosis. The MN of mononuclear cells are not often analysed but were of particular interest. The most prevalent research into MN has involved binucleated cells (BN cells) that have only completed one mitotic event. In additional, in samples of three-, four-, five- and polynuclear cells were found that had completed more than one mitotic event from the start of their cultivation.

The rate of cell proliferation was estimated by a proliferation index (shown below), according to an international protocol. According to current studies, the proliferation index should not exceed 2.0 in healthy people and is defined in the literature in two ways. In this study we used the following formula:

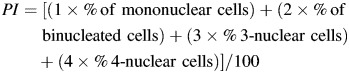



The median proliferation index of the radon-exposed group was significantly increased compared to the control group (2.04 and 1.97, respectively; *P* < 0.01 determined by the Mann–Whitney test). In addition, the radon-exposed group exhibited a lower number of mono- and binucleated cells and an increased number of polynucleated cells (Table 4). The Spearman correlation coefficient between proliferation index and age did not reveal a significant relationship (*R* = − 0.14, *P* = 0.28 in the exposed group; *R* = − 0.18, *P* = 0.18 in the control group). Based on our observations, we propose that the increased proliferation serves as a compensatory mechanism to counter genotoxic effects from radon.

**Table 4.** The value of proliferation index (PI) and cell spectrum

	*PI*, M ± m	Total cells, %		
		mononucleated, M ± m	binucleated, M ± m	polynucleated, M ± m
Exposed group	2.04* ± 0.002	26.0 ± 0.08	25.3* ± 0.08	40.8* ± 0.02
Control group	1.97 ± 0.002	27.7 ± 0.14	26.8 ± 0.08	38.0 ± 0.04

Significant difference between groups: *P < 0.01.

Binucleated lymphocytes that had completed one mitotic division were counted and analysed for DNA damage; such widely used methods are considered extremely useful for ecological and genetic research. In this study, 1000 cells were counted and categorised, based upon the presence of MN, NPBs and protrusions.

The control group displayed an increased frequency of cells with NBUDs, but this change was insignificant. No significant difference was determined for the number of cells containing NPBs (Table 5).

**Table 5.** Characteristics of binucleated lymphocytes

	Total number of micronuclei	Cells with MN (%), M ± m	Cells with NPBs (%), M ± m	Cells with NBUDs (%), M ± m
Exposed group	358*	0.6* ± 0.005	0.39 ± 0.01	0.79 ± 0.02
Control group	202	0.3 ± 0.003	0.33 ± 0.01	1.23 ± 0.02

Significant difference between groups: *P < 0.001.

**Fig. 1.**
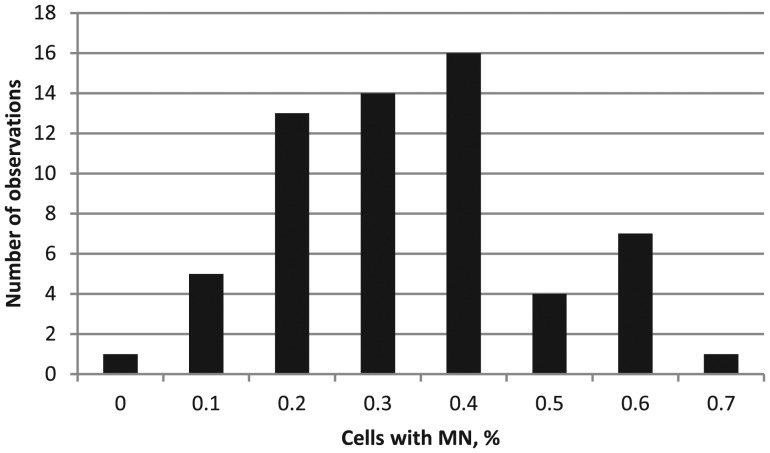
Distribution of cells with MN (control group).

**Fig. 2.**
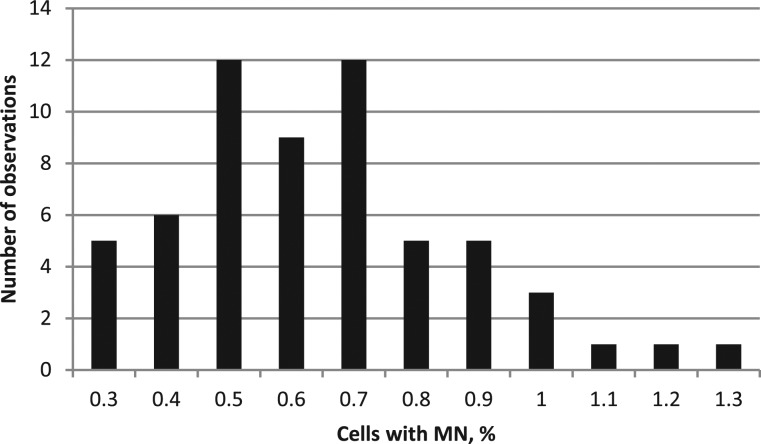
Distribution of cells with MN (exposed group).

The frequency of cells with MN is a key indicator of the extent of DNA damage due to exposure to various genotoxicants, including radon. A distribution analysis of the frequency of cells with MN was conducted using the Shapiro–Wilk test and yielded an index of 0.9496 (*P* = 0.015) in the control group (Fig. 1) and 0.9497 (*P* = 0.0151) in the exposed group (Fig. 2). In the exposed group, 0.6% of cells had MN (per 1000 cells, with a range of values from 0.3–1.3) while in the control group, 0.3% of cells had MN (per 1000 cells, with a range of values from 0–0.7); this difference was statistically significant (*P* < 0.001) (Table 5). In the exposed group, 52 people had a higher frequency of cells with MN (per 1000 cells) than their counterparts in the control group. We calculated the Spearman correlation coefficient for each of these indices, between age and the frequency of chromosomal aberrations. In the control group, a small positive yet significant correlation was found between the number of cells with MN and the frequency of chromosomal aberrations (*R* = 0.298, *P* = 0.021). In addition, a significant increase in the frequency of binucleated lymphocytes with MN in females compared with males (4.31/1000 vs 3.7/1000, *P* = 0.044) was observed in the control group, consistent with data from previous studies [16]. In the exposed group, no statistically significant differences were observed.

**Fig. 3.**
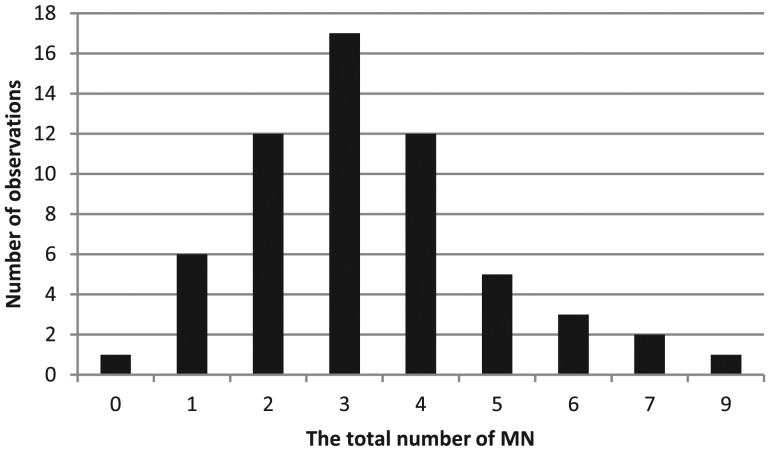
Distribution of the total number of MN (control group).

**Fig. 4.**
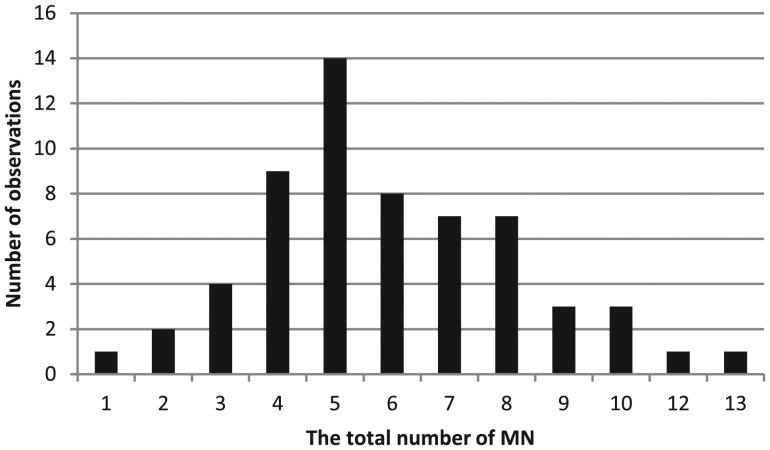
Distribution of the total number of MN (exposed group).

A significant increase (*P* < 0.001) in the total number of micronuclei was found in the radon-exposed group compared with the control group (358 and 202, respectively) (Table 5). A distribution analysis of the frequency of cells with MN was conducted using the Shapiro–Wilk test and yielded an index of 0.9363 (*P* = 0.0037) in the control group (Fig. 3) and 0.9607 (*P* = 0.0411) in the exposed group (Fig. 4).

The majority of binucleated lymphocytes in both the control and the exposed groups had only one MN. However, seven cells exhibited two MN and one cell exhibited three MN in the control group, while in the exposed group 12 cells exhibited two MN and three cells exhibited three MN.

According to prior studies [16], increase in the frequency of MN is proportional to age. In our study, no significant correlations were found between these parameters in either the control or the experimental groups (*R* = − 0.134, *P* = 0.31 and *R* = − 0.074, *P* = 0.53, respectively). These results can be explained by the small sample size and the small spread in the age of the subjects. In our study, the spread of age was only 7 years old (8–18 years) fitted into just one of the age cohorts reported by Vral *et al*. [16].

No differences were noted between individuals of different ethnicities.

Because NPBs are a result of chromosomal aberrations and the formation of dicentric and polycentric chromosomes, the comparison of the values from the micronucleus test with those obtained from the analysis of chromosomal aberrations was of special interest in our study. However, the calculation of the correlation coefficient showed no significant difference between these parameters. In the control group, *R* was − 0.071, *P* = 0.59, while in the exposed group, *R* was 0.055, *P* = 0.68. This result can be explained by the small number of cells with bridges and dicentric chromosomes.

## CONCLUSION

The DNA of people living in areas with high radon concentrations may be affected by substantial genotoxicity. This was reflected in our study by an increased frequency of cells with micronuclei (MN), particularly in binucleated peripheral blood lymphocytes (0.6% of cells with MN in the exposed group vs 0.3% in the control, *P* < 0.001). MN were observed in cells that had progressed through more than one mitotic event. In addition, the exposed group also showed a significant increase in the total number of micronuclei.

The proliferation index, which characterises the rate of cell division, was also higher in the exposed group (2.04 vs 1.97). This increase might have been caused by a compensatory mechanism to promote a more rapid renewal of the proliferative pool under the genotoxic effects of radon.

A low yield of NPBs and NBUDs prevented an evaluation of a correlation with radon concentration. Further studies are required to address this. The CBMN is a convenient and effective method for assessing the genome damage caused by genotoxicants, and it can be used in ecological and genetic studies as a biological screening test.

## FUNDING

This work was supported by the Russian Foundation for Basic Research (RFBR) (grant 12-04-32218-mol_a) and by the State Contract (16.512.11.2062).
